# Evaluation of feed withdrawal prior to marketing to improve carcass yield and reduce feed cost in finishing pigs

**DOI:** 10.1093/tas/txaf046

**Published:** 2025-04-16

**Authors:** Hilario M Cordoba, Joel M DeRouchey, Jason C Woodworth, Mike D Tokach, Robert D Goodband, Jordan T Gebhardt

**Affiliations:** Department of Animal Sciences and Industry, College of Agriculture, Kansas State University, Manhattan, KS 66506-0201, USA; Department of Animal Sciences and Industry, College of Agriculture, Kansas State University, Manhattan, KS 66506-0201, USA; Department of Animal Sciences and Industry, College of Agriculture, Kansas State University, Manhattan, KS 66506-0201, USA; Department of Animal Sciences and Industry, College of Agriculture, Kansas State University, Manhattan, KS 66506-0201, USA; Department of Animal Sciences and Industry, College of Agriculture, Kansas State University, Manhattan, KS 66506-0201, USA; Department of Diagnostic Medicine/Pathobiology, College of Veterinary Medicine, Kansas State University, Manhattan, KS 66506-0201, USA

**Keywords:** carcass characteristics, carcass yield, feed withdrawal, finishing pig

## Abstract

Fasting pigs prior to harvest benefits food safety and pork quality. Studies have examined the effect of feed withdrawal prior to marketing applied on groups of pigs marketed at one time but not across multiple marketing events. Therefore, this study determined the effects of three feed withdrawal times before the first and final marketing event on pig performance, carcass traits, and economic return. A total of 695 finishing pigs (117.6 ± 1.06 kg) were allotted in a randomized complete block design and assigned to 1 of 3 treatments (24 pens per treatment; 9 to 10 pigs per pen). Treatments consisted of 12, 18, or 24 h (including transportation and lairage) of feed withdrawal prior to harvest to determine its effects on hot carcass weight (HCW), carcass yield, and economic costs vs. benefits of pigs marketed at two different marketing events (3 heaviest pigs per pen marketed 14-d prior to the final marketing of all remaining pigs). There were no differences in growth performance during the 14-d period between marketing events. However, pigs with 24 h of feed withdrawal prior to harvest had lower (*P* < 0.05) live BW at the first marketing event and at both events combined than pigs with 12 h of feed withdrawal. For carcass responses, pigs at the final marketing with 12 h of feed withdrawal prior to harvest had increased (*P* < 0.05) HCW compared to pigs with 24 h of feed withdrawal, leading to a tendency (*P* = 0.055) for increased HCW overall for the combined marketing events (0.5 kg heavier). When evaluating carcass yield using live weights at 24 h prior to harvest, pigs in the final marketing group with 12 h of feed withdrawal prior to harvest had greater yield (*P* < 0.05) than pigs marketed with 24 h of feed withdrawal; however, using live weights 12 h prior to harvest to calculate yield, pigs with 24 h of feed withdrawal prior to harvest had increased yield (*P* < 0.05) compared to pigs marketed with 12 h of feed withdrawal for both marketing events. There were no differences in backfat, loin depth, and percentage lean between treatments. Feed consumed and cost were reduced (*P* < 0.05) with 24 h treatment compared to 12 h in the overall period. In conclusion, withholding feed at the first marketing event did not impact pigs’ growth performance or HCW. However, carcass yield was affected by the feed withdrawal time prior to harvest, with greater HCW for pigs with 12 h compared to 24 h of feed withdrawal at the final marketing event.

## Teaser

Withholding feed before harvest is beneficial to reduce in-barn feed usage on the day of marketing and benefits pork meat quality and safety. However, if feed is withheld for more than 18 h before harvest, hot carcass weight can decrease.

## INTRODUCTION

Finishing pigs inherently experience periods of feed deprivation during transport and lairage. Feed withdrawal prior to transportation to a processing plant implies reduced feed intake on the day of marketing and thereby reduces feed cost per pig (Kephart and Mills, 2005). It was also suggested that feed withdrawal up to 24 h improved meat color and water-holding capacity due to a decrease in muscle glycolytic potential (Bertol et al., 2005). Fasting also reduces gut fill, which ultimately reduces waste at the abattoir (Eikelenboom et al., 1991) and improves food safety by lowering the risk of carcass contamination with pathogenic microorganisms for humans (Fernandez, 2021).

Frobose et al. (2014) observed a linear reduction in hot carcass weight (HCW) with 8, 24, 26, and 48 h (including a total of 8 h of loading, transportation, and lairage) of feed removal prior to harvest, which resulted in a linear reduction of feed consumed the day of marketing as feed removal time increased. The HCW reduced 0.3 kg when feed withdrawal time was extended from 8 to 24 h, with reductions being 2 kg or greater at or above 36 h of feed removal. These data were based on the last marketing of pigs, but to our knowledge no data is available on feed removal for the first group of pigs to be marketed (topped). With current feeder designs utilizing quick adjust settings, feeders could be closed to prevent pigs from eating prior to topping events and then reset after pigs are marketed to supply feed to the remaining pigs in the pen. Thus, these first marketed pigs could benefit from a short feed removal prior to shipment.

This study aimed to determine the effects of feed withdrawal prior to marketing on pig performance, carcass traits, and economic return over two marketing events. We hypothesized that increased withholding feed time prior to marketing would reduce HCW and increase carcass yield, affecting economic outcomes. Additionally, we expected the feed withdrawal at the first marketing event would not be long enough to decrease the performance of the pigs that remain until the final marketing event.

## MATERIALS AND METHODS

### General

The Kansas State University Institutional Animal Care and Use Committee approved the protocol used in this experiment. This study was conducted at the Kansas State University Swine Teaching and Research Center in Manhattan, KS. The facility was totally enclosed and environmentally regulated, containing 36 pens in each barn. Each pen was equipped with a two-hole dry single-sided feeder (Jumbo Feeder, Farmweld, Teutopolis, IL) and a 1-cup waterer. Pigs were stocked at a floor space of approximately 0.65 m^2^ per pig excluding feeder footprint area. Pens were equipped with adjustable gates to allow space allowances per pig to be maintained if a pig died or was removed from a pen during the experiment. Pens were located over a completely slatted concrete floor with a 1.22-m pit underneath for manure storage. A robotic feeding system (FeedPro; Feedlogic Corp., Wilmar, MN) was used to deliver and record daily feed additions to each individual pen.

### Animals and Diets

A total of 695 pigs (600 × 241, DNA; initially 117.6 ± 1.06 kg) were used in a 14-d study. Pigs were housed in mixed gender pens with 9 or 10 pigs per pen and 24 pens per treatment. Pens of pigs were group-weighed 5-d prior to the start of the study and assigned to 1 of 3 treatments in a randomized complete block design with initial pen average weight as a blocking factor. Treatments consisted of none, 6, or 12 h of feeder closure prior to pig removal from pens at both the first and final (2 weeks after the first) marketing events to achieve approximately 12, 18 and 24 h of total feed withdrawal prior to harvest at the processing plant. Pigs were fed a corn-soybean meal-based diet that was formulated to meet or exceed nutrient requirements for pigs weighing 110 to 130 kg (NRC, 2012).

Pigs and feeders were weighed at the first and final marketing event at 8:30 h and 20:30 h to determine feed intake and individual BW within the marketing day, and to calculate ADG, ADFI, and G:F. Feed intake measurements also accounted for feed remaining in the pan after feeders were closed. The remaining feed in the pan was disposed after weighing feeders. Feeders from treatments with 18 and 24 h of feed withdrawal prior to harvest were closed at 14:30 h and 8:30 h, respectively, whereas feeders from the treatment with 12 h of feed withdrawal prior to harvest remained open. The total time without access to feed for all the treatments included 12 h of transportation and lairage time at the packing plant. Pigs had access to water before loading out and at the packing plant until slaughter time but not during transportation. Pigs were loaded out of barns at 20:30 h at each marketing event. At the first marketing event, the 3 heaviest pigs in each pen were selected for marketing and pens were adjusted to maintain stock density at 0.65 m^2^ per pig.

Pigs were individually identified with an electronic tag and a tattoo for carcass data collection, transported to a USDA-inspected packing plant (Triumph Foods, St. Joseph, MO,) for harvest at 8:30 h the following day. Carcass measurements included HCW, loin depth, backfat, and percentage lean. Percentage lean was calculated from a plant proprietary equation. Carcass yield was calculated by dividing the pen average HCW by the individual live weight recorded in the morning (24 h before harvest and before feed withdrawal) and in the evening (12 h before harvest after feed withdrawal treatments) of each marketing event. Feed cost was calculated using feed intake multiplied by a feed cost of $0.276/kg.

### Statistical Analysis

Data were analyzed as a randomized complete block design for one-way ANOVA using the lmer function from the lme4 package in R (version 4.4.1 (2021-08-10), R Foundation for Statistical Computing, Vienna, Austria) with pen considered as the experimental unit, initial weight as a blocking factor, and treatment as a fixed effect. The overall marketing data were analyzed by calculating a weighted average of the first and final marketing event for each response. The BW collected 24 h before harvest was used as covariate for analysis of HCW. Hot carcass weight was used as a covariate for backfat, loin depth, and percentage lean. Results were significant if *P*-values were ≤ 0.05 and considered tendencies if *P*-values were ≤ 0.10.

## RESULTS

For pig BW recorded 24 h before harvest, there were no differences (*P* > 0.10) between treatments at both marketing events and overall. However, at time of loadout (12 h before harvest), pigs enrolled on the 24 h of feed withdrawal prior to harvest treatment were lighter (*P* < 0.05) than those on the 12 h withdrawal prior to harvest, both at first marketing and for overall marketing data, but not for the final marketing event (Table 1). This was a result of pigs having different weight gain on each marketing day, with pigs on 12 h of feed withdrawal prior to harvest having greater gain (*P* < 0.05) than the other two treatments.

**Table 1.** Effects of withholding feed prior to harvest on growth performance^1^

	Feed withdrawal time before harvest, h^2^				
Item	12	18	24	SEM	*P* =
BW, kg					
24 h before harvest					
First marketing	126.4	125.6	124.7	1.19	0.334
Final marketing	127.3	127.2	127.1	1.21	0.994
Overall^3^	126.9	126.7	126.5	1.08	0.850
12 h before harvest (at time of loading on the truck)					
First marketing	127.4^a^	125.2^ab^	123.9^b^	1.10	0.003
Final marketing	127.6	127.1	125.5	1.27	0.153
Overall^3^	127.4^a^	126.4^ab^	125.1^b^	1.11	0.020
BW change, kg/pig^4^					
First marketing	1.13^a^	-0.49^b^	-0.86^b^	0.444	< 0.001
Final marketing	0.31^a^	-0.15^a^	-1.67^b^	0.200	< 0.001
Overall^3^	0.57^a^	-0.26^b^	-1.43^c^	0.222	< 0.001
Overall (d 0 to 14)					
ADG, kg	0.99	0.98	0.95	0.032	0.528
ADFI, kg	2.84	2.84	2.85	0.029	0.997
G:F	0.35	0.35	0.34	0.011	0.439

^1^A total of 695 mixed sex pigs (initial BW 117.6 ± 1.06 kg) were used in a 14-d trial with 24 replications per treatment.

^2^The 3 treatments consisted of none, 6, or 12 h of feeder closure prior to loading on the truck at both the first marketing event (2 weeks before final marketing) and final marketing event to achieve 12, 18 and 24 h, respectively, of total feed withdrawal prior to harvest.

^3^Weighted average from the first and final marketing.

^4^Body weight difference between the 24 h and 12 h before harvest weight determination.

At the first marketing event, pigs BW loss was greater (*P* < 0.05) on 18 and 24 h of feed withdrawal prior to harvest compared to pigs on 12 h of feed withdrawal prior to harvest, but no differences (*P* ≥ 0.10) were observed between the 18 and 24 h feed withdrawal strategies. At the final marketing event, pigs on 24 h feed withdrawal prior to harvest had increased (*P* < 0.05) BW loss compared to the 12 and 18 h feed withdrawal strategies, but no differences were observed (*P* ≥ 0.10) between the 12 and 18 h feed withdrawal treatments. However, when both marketing events were combined, pigs had greater (*P* < 0.05) BW loss as the time feed withdrawal prior to harvest increased.

For pig performance during the 14-d period between the first and final marketing event, there was no differences (*P* ≥ 0.10) for any growth response criteria.

For carcass characteristics, there were no differences (*P* > 0.10) in HCW at the first marketing event. However, pigs at the final marketing event that had 12 h of feed withdrawal prior to harvest had increased (*P* < 0.05) HCW compared to those with 24 h of withdrawal prior to harvest (Table 2). When considering both marketing events, there was a tendency (*P* = 0.055) for a treatment effect with pigs having 12 h feed withdrawal prior to harvest having a 0.5 kg heavier HCW than those with 24 h feed withdrawal prior to harvest. When evaluating carcass yield using live weights for all pigs obtained 24 h prior to harvest, no differences (*P* > 0.10) were observed at the first marketing event, but pigs in the final marketing group with 12 h of feed withdrawal before harvest had increased carcass yield (*P* < 0.05) compared to pigs with 24 h of feed withdrawal before harvest. However, when evaluating carcass yield using live weights obtained at load out (12 h prior to harvest) at the first marketing event, pigs with 24 h of feed withdrawal prior to harvest had increased (*P* < 0.05) carcass yield compared to only the pigs with 12 h feed withdrawal prior to harvest. At the final marketing event and overall, pigs with 24 h of feed withdrawal prior to harvest had increased yield (*P* < 0.05) compared with the other two treatments. There were no differences in backfat depth, loin depth, and percentage lean observed between treatments (*P* > 0.10).

**Table 2.** Effects of withholding feed prior to harvest on carcass characteristics^1^

	Feed withdrawal time prior to harvest, h^2^				
Item	12	18	24	SEM	*P* =
Carcass characteristics					
HCW, kg^3^					
First marketing	92.4	92.2	92.6	0.41	0.573
Final marketing	93.6^a^	93.3^ab^	92.8^b^	0.17	0.010
Overall^4^	93.2	93.0	92.7	0.20	0.055
Carcass yield, %					
24 h before harvest					
First marketing	73.8	73.6	73.8	0.30	0.686
Final marketing	73.4^a^	73.2^ab^	72.9^b^	0.10	0.010
Overall^4^	73.5	73.3	73.1	0.20	0.099
12 h before harvest					
First marketing	73.1^b^	73.8^ab^	74.3^a^	0.30	0.012
Final marketing	73.2^b^	73.3^b^	73.8^a^	0.10	0.003
Overall^4^	73.2^b^	73.5^b^	73.9^a^	0.10	< 0.001
Backfat, mm^5^					
First marketing	13.5	13.3	13.2	0.21	0.572
Final marketing	13.9	13.7	14.1	0.17	0.259
Overall^4^	13.7	13.6	13.8	0.14	0.449
Loin depth, mm^5^					
First marketing	65.9	67.0	66.1	0.55	0.945
Final marketing	65.5	65.5	64.8	0.36	0.254
Overall^4^	65.6	65.7	65.2	0.31	0.492
Lean, %^5^					
First marketing	56.0	56.1	56.2	0.20	0.703
Final marketing	55.7	55.8	55.5	0.10	0.136
Overall^4^	55.8	55.9	55.7	0.10	0.372
Feed consumed, kg/pig^6^					
First marketing	1.56^a^	0.80^b^	0.09^c^	0.096	< 0.001
Final marketing	1.12^a^	1.19^a^	0.14^b^	0.079	< 0.001
Overall^4^	1.26^a^	1.07^a^	0.12^b^	0.069	< 0.001
Feed cost, $/pig^7^					
First marketing	0.43^a^	0.22^b^	0.03^c^	0.026	< 0.001
Final marketing	0.31^a^	0.33^a^	0.04^b^	0.022	< 0.001
Overall^4^	0.35^a^	0.29^a^	0.03^b^	0.019	< 0.001

^1^A total of 695 mixed sex pigs (initial BW 117.6 ± 1.06 kg) were used in a 14-d trial with 24 replications per treatment.

^2^The 3 treatments consisted of none, 6, or 12 h of feeder closure prior to loading on the truck at both the first marketing event (2 weeks before final marketing) and final marketing event to achieve 12, 18 and 24 h, respectively, of total feed deprivation prior to harvest.

^3^24 h BW before harvest was used as a covariate for analysis of HCW.

^4^Weighted average from the first and final marketing.

^5^HCW was used as a covariate for analysis of backfat, loin depth, and percentage lean.

^6^Feed consumed the day of marketing (24 h and 12 h before harvest).

^7^Feed consumed between 24 h and 12 h before harvest multiplied by a diet cost of $0.276/kg.

For feed consumed and feed cost, in the first marketing event, pigs marketed with 12 h feed withdrawal prior to harvest had increased (*P* < 0.05) feed consumption and feed cost compared to those with 18 h feed withdrawal, with pigs marketed with 24 h feed withdrawal prior to harvest having the lowest (*P* < 0.05) feed consumption and feed cost. This represents 1.47 and 0.71 kg less feed consumed per pig between the 12 and 18 h treatment, respectively, in comparison with the 24 h treatment. On the final marketing event and overall, feed consumed and feed cost per pig were increased (*P* < 0.05) for pigs marketed with 12 or 18 h of feed withdrawal before harvest compared to those with 24 h feed withdrawal prior to harvest, which accounts for a total feed intake reduction of 1.14 kg between the 12 and 24 h treatments, representing $0.32 per pig in the overall period.

## DISCUSSION

Reducing accessibility to feed prior to harvest decreases feed consumed during the marketing day resulting in reduced pigs’ body weight due to emptying the gastrointestinal tract (Driessen et al., 2020). This was demonstrated by Frobose et al. (2014), who used 8, 24, 36, and 48 h of feed withdrawal prior to harvest in a commercial facility and observed a linear reduction in feed consumed during the marketing day as feed withdrawal time increased, resulting in a linear reduction in HCW and carcass yield. In our study, feed withdrawal was established to understand the effect of carcass traits and economic responses using 12, 18, and 24 h of feed restriction prior to harvest where transportation and lairage time were considered. In previous studies, feed withheld for 24 h prior to harvest resulted in lighter pigs than those that had accessibility to feed for 12 h prior to harvest including transportation and lairage time (Jones et al., 1985; Bertol et al., 2005; Panella-Riera et al., 2012). The same response was observed in our study, where pigs’ BW decreased as feed withdrawal time increased from 12 to 24 h in the first marketing event and when overall data was summarized. However, in the final marketing event, even though there was a difference of 2.1 kg of BW between pigs without access to feed for 24 h compared to 12 h, the difference was not significant. The reason for these results might be related to the season of the year and the procedures during each marketing day. Daily temperature was elevated during the final marketing day as compared to the first marketing event, which likely reduced feed intake of pigs with accessibility to feed before loading out on the final marketing day.

Another difference between our study and those observed by others may be that we maintained 0.65 m^2^ per pig for the remaining 14 d from the first and last marketing events by adjusting available floor space. In a commercial facility, after removing the 10 to 20% heaviest pigs in a pen, floor and feeder space allowance is increased which helps to avoid growth restriction. This was demonstrated by Flohr et al. (2016) who used three marketing events providing 0.65 m^2^ per pig and removed pigs as space became constrained to not restrict growth based on Gonyou et al. (2006) allometric equations. In our study, after removing the 3 heaviest pigs at the first marketing event, the front gate of each pen was adjusted to keep the initial 0.65 m^2^ per pig. This practice allows us to see the effect of feed withdrawal and avoid having a space allowance effect.

Morrow et al. (2002) conducted a study marketing finishing pigs at three different times to see the effect on carcass composition, but they did not measure growth performance of the pigs between each marketing event. To our knowledge, there is limited data in the literature showing results for growth performance between marketing events in the same group of pigs. In this study, our results showed that reducing in-barn feed accessibility at the first marketing event for up to 12 hours did not affect the performance of the pigs remaining in the pens for the next 14 d until marketed.

The HCW in the first marketing was not different between the three durations of feed withdrawal. These results were not expected because feeders were shut down to accomplish the feed withdrawal time and the feed intake decreased as the time of feed withdrawal increased. However, at the final marketing event, the results showed the feed restriction response in gain and HCW. Pigs having access to feed increased gain during the marketing day, and that was reflected in the HCW results. When results observed at the first and final marketing event were combined, a tendency for a marginal increase in HCW of 0.5 kg was observed when pigs had access to feed for 12 h before being harvested in comparison to those without for 24 h prior to harvest. These results agree with results of Fàbrega et al. (2019), Frobose et al. (2014), and Faucitano et al. (2006) where HCW decreased as the time of feed withdrawal prior to harvest increases.

In this study, pigs were weighed individually before and after the fasting period. When carcass yield was calculated using the live weights before the period of feed withdrawal, carcass yield increased when pigs had 12 h of feed withdrawal compared to those with 24 h at the final marketing event. This is a result of HCW loss after the feed withdrawal period prior to harvest. In contrast, when carcass yield was calculated using the BW recorded after the fasting period, carcass yield increased for those pigs with 24 h of feed withdrawal compared to those with 12 h at the final marketing event and overall. This is a result of the gut fill at the time BW was recorded which affected carcass yield. Similar results were observed by Frobose et al. (2014) when feed was withdrawn for 8, 24, 36, and 48 h prior to harvest, where carcass yield increased as the time without access to feed increased. Nonetheless, these results were different from those observed by Kephart and Mills (2005), Jones et al. (1985), Eikelenboom et al. (1991) and Faucitano et al. (2006) who applied between 0 to 48 h of feed withdrawal and did not find differences in carcass yield over 24 h of feed withdrawal.

Backfat depth, loin depth, and percentage of lean were analyzed to determine the effect of feed withdrawal prior to harvest. Some authors Morrow et al. (2002) and Leheska et al. (2003) reported no differences in those carcass traits when having 12 to 48 h of feed withdrawal. However, with the decrease in HCW observed in past literature and with current fast growth genetics (Frobose et al., 2014), it is important to evaluate if feed withdrawal also affects carcass characteristics. The results of our study showed that feed withdrawal time used did not affect backfat, loin depth, and percentage of lean at any marketing event and overall.

Frobose et al. (2014) observed savings of 5 kg of feed per pig during the time prior to marketing when feed was withdrawn for 48 h compared to 8 h. A linear response on feed savings was observed in our study as the time of feed withdrawal increased. At the first marketing event, pigs without access to feed for 24 h and 18 h reduced feed consumption by 1.47 and 0.71 kg, respectively, compared to pigs withheld for 12 h. However, at the final marketing event the feed intake results were different. There was a reduction in feed consumption for pigs with 24 h of feed withdrawal compared to the 12 and 18 h treatments, but no differences in feed intake between pigs with feed withdrawn for 12 and 18 h, even though pigs had 6 h more of feed access. This could be a result of the high summer temperatures during the second marketing day, reducing the desire for pigs to consume feed. The feed cost calculation is directly tied to the feed intake response. Therefore, it is necessary to consider if the savings in feed cost will offset the losses in HCW. The level of HCW loss will be dependent on the total time without access to feed from in-barn feeder closure and including transportation and lairage period.

These data showed that after removing the 3 heaviest pigs from each pen in the first marketing event, there were no effects of feed withdrawal at different times prior to harvest on growth performance of the pigs that stayed until the end of the study nor was there any effect on HCW, carcass yield, and economics for pigs marketed at the first marketing event. However, at the final marketing event and overall, differences in HCW, carcass yield, and economics were observed. These data suggest that feed withdrawal time greater than 18 h reduces HCW. Therefore, we conclude that reduced feed cost will have to offset potential losses in HCW when times of feed withdrawal exceed 18 h prior to harvest. In addition, the time without access to feed should consider the duration of feed withdrawal as well as transportation and lairage times. Therefore, knowing the duration of transportation and lairage is crucial to properly apply feed withdrawals prior to harvest.
